# Burst sine wave electroporation (B-SWE) for expansive blood–brain barrier disruption and controlled non-thermal tissue ablation for neurological disease

**DOI:** 10.1063/5.0198382

**Published:** 2024-05-30

**Authors:** Sabrina N. Campelo, Zaid S. Salameh, Julio P. Arroyo, James L. May, Sara O. Altreuter, Jonathan Hinckley, Rafael V. Davalos, John H. Rossmeisl

**Affiliations:** 1 Virginia Tech-Wake Forest School of Biomedical Engineering and Sciences, Virginia Tech, 325 Stanger St, Blacksburg, Virginia 24061, USA; 2 Wallace H. Coulter Department of Biomedical Engineering at Georgia Institute of Technology and Emory University, Atlanta, Georgia 30332, USA; 3 Department of Small Animal Clinical Sciences and Animal Cancer Care and Research Center, Virginia-Maryland College of Veterinary Medicine, Virginia Tech, Blacksburg, Virginia 24061, USA

## Abstract

The blood–brain barrier (BBB) limits the efficacy of treatments for malignant brain tumors, necessitating innovative approaches to breach the barrier. This study introduces burst sine wave electroporation (B-SWE) as a strategic modality for controlled BBB disruption without extensive tissue ablation and compares it against conventional pulsed square wave electroporation-based technologies such as high-frequency irreversible electroporation (H-FIRE). Using an *in vivo* rodent model, B-SWE and H-FIRE effects on BBB disruption, tissue ablation, and neuromuscular contractions are compared. Equivalent waveforms were designed for direct comparison between the two pulsing schemes, revealing that B-SWE induces larger BBB disruption volumes while minimizing tissue ablation. While B-SWE exhibited heightened neuromuscular contractions when compared to equivalent H-FIRE waveforms, an additional low-dose B-SWE group demonstrated that a reduced potential can achieve similar levels of BBB disruption while minimizing neuromuscular contractions. Repair kinetics indicated faster closure post B-SWE-induced BBB disruption when compared to equivalent H-FIRE protocols, emphasizing B-SWE's transient and controllable nature. Additionally, finite element modeling illustrated the potential for extensive BBB disruption while reducing ablation using B-SWE. B-SWE presents a promising avenue for tailored BBB disruption with minimal tissue ablation, offering a nuanced approach for glioblastoma treatment and beyond.

## I. INTRODUCTION

Glioblastoma stands as the most prominent malignant brain tumor, constituting over 50% of all malignant brain tumors. Despite advancements in standard of care therapies, which currently include surgical resection, chemotherapy, and/or radiotherapy, the prognosis remains grim, with a five-year survival rate of approximately 7%.^1^ With the addition of the chemotherapy drug temozolomide, median survival has increased from about 12 to 14.5 months.^2^ Much of this poor prognosis is attributed to the presence of the blood–brain barrier (BBB), which serves to screen out toxins and pathogens in the circulatory system from entering the central nervous system. However, its mechanism oftentimes impedes large therapeutics from entering the brain parenchyma for effective tumor targeting. Consequently, there is a pressing need for innovative methods to address the treatment of malignant brain tumors, such as glioblastoma, and overcome the existing limitations in the current standard of care.

Irreversible electroporation (IRE) is a therapeutic approach utilizing pulsed electric fields (PEF) to induce cell death in soft tissues.^3^ Unlike thermally based ablation modalities, which lack specificity in causing tissue death and may lead to neurological deficits, such as alterations in cognitive function, motor skills, sensory perception, or other neurological impairments, IRE's non-thermal cell death mechanism emerges as a preferred option for treating tumors located in close proximity to blood vessels, nerves, and acellular architecture. More recently, a shift toward higher frequency IRE waveforms, termed high-frequency irreversible electroporation (H-FIRE), has been explored to reduce neuromuscular stimulation and treatment-induced impedance changes, in turn allowing for more predictable treatment volumes.^4^ In addition to its application in soft tissue ablation, H-FIRE has been shown to focally and transiently permeate the endothelial lining of the BBB for up to 72 h, allowing for the passage of larger impermeable drugs and chemotherapeutics during this time.^5–7^

Until now, conventional electroporation protocols have predominantly employed monopolar and bipolar square-shaped electrical pulses to permeabilize and ablate cells. The nearly discrete nature of square-shaped pulses ensures that 100% of the delivered energy is targeted toward prioritizing ablation field coverage around a specific target. As electroporation is a threshold-type phenomenon, being at or above the specific threshold for a certain amount of time is critical for inducing the desired electroporation-based biophysical responses (BBB disruption, reversible electroporation, tissue ablation, etc.). Although sinusoidal-shaped waveforms have been investigated in previous studies, evidence has supported the idea that square-shaped wave pulses are more effective in inducing electroporation when compared to sinusoidal-shaped waveforms or other waveforms with slower rise and fall times.^8,9^ In the case of sinusoidal-shaped waveforms, the electric field amplitude is below the required ablation field threshold for a given amount of time, even when the pulse is on, implying that the energy is being used on other low-field processes such as electrophoresis, electrolysis, and Joule heating rather than tissue ablation. Nevertheless, it is important to note that these secondary effects persist even with square-shaped waveforms.

This study proposes a strategic utilization of the extended rise and fall times associated with burst sine wave electroporation (B-SWE). BBB disruption operates as a low-field phenomenon with reported electric field thresholds (EFT) below 216 V/cm which is significantly below the high-field thresholds required for irreversible electroporation (noted at fields exceeding 725 V/cm in rat gliomas).^5,10^ The transient nature of the applied sinusoidal-shaped field may allow one to capitalize on the gap between these thresholds to modulate the extent of BBB disruption relative to the non-thermal tissue ablation volumes. Herein, we utilize an *in vivo* rodent model to leverage the sub-ablation field strength duration of the sinusoidal-shaped burst to induce diffuse regions of BBB disruption, while controlling the extent of tissue ablation, in comparison to conventional square-shaped H-FIRE bursts.

## II. RESULTS AND DISCUSSION

To compare the biophysical effects of B-SWE and H-FIRE on brain tissue, a healthy *in vivo* rat brain model was utilized. Outcomes investigated included BBB disruption volumes, tissue ablation volumes, and extent of neuromuscular contractions.

### A. Equivalent B-SWE and H-FIRE Waveforms

**FIG. 1.**
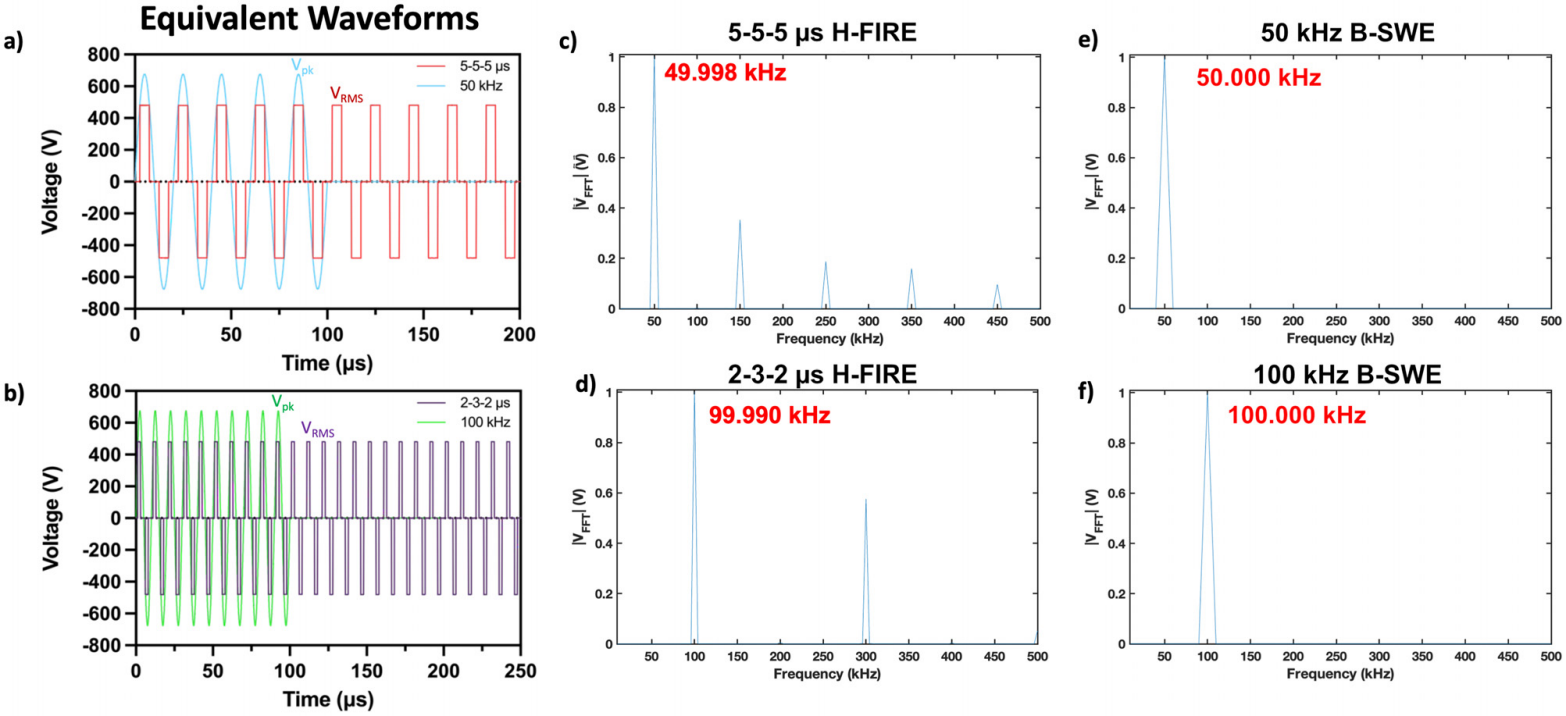
Equivalent sinusoidal-shaped waveforms can be derived from square-shaped waveforms by matching equivalent areas under the curve. (a) and (b) Root mean square calculations are used to derive the required applied electric potential needed for applying an equivalent voltage. Peak voltages ( $V_{pk}$) are applied for sinusoidal-shaped waveforms and root mean square voltages ( $V_{\mathrm{RMS}}$) for square-shaped waveforms with equivalent energized burst times (100 *μ*s). (c)–(f) Waveform frequencies are matched through Fourier transform analysis by transforming the time domain square-shaped waveform into the frequency domain. A normalized frequency spectrum of the 5-5-5 *μ*s square-shaped waveform is shown with a maximum peak magnitude similar to the 50 kHz sine wave, while the normalized frequency spectrum of the 2-3-2 *μ*s square-shaped waveform is shown with a maximum peak magnitude similar to the 100 kHz sine wave.

To facilitate a direct comparison between sinusoidal and square-shaped waveforms, paired protocols ensuring equivalent frequency and induced electric field components were chosen and shown below in Fig. 1. For the B-SWE protocols, a waveform frequency of 50 kHz was chosen based on its heavily studied presence in the literature for inducing clinically relevant H-FIRE ablations,^10^ while 100 kHz was chosen as previous literature has indicated that radio-frequency pulses above 100 kHz completely eliminate muscle contractions.^11^ The 50 and 100 kHz B-SWE waveforms were delivered at a sinusoidal peak voltage $\left(V_{pk}\right)$ of 680 V, while their equivalent 5-5-5 *μs* and 2-3-2 *μs* H-FIRE waveforms were delivered at a root mean squared voltage ( $V_{\mathrm{RMS}}$) equivalent of 480 V, respectively. The nomenclature for H-FIRE waveforms follows *PulseOn-PulseDelay-PulseOn*. All waveforms were delivered for a total burst energized time of 100 *μs*, where energized time refers to the duration that the waveform is in the “PulseOn” position.

While the fast Fourier transform (FFT) algorithm was used to validate the frequency behavior of our square-shaped waveforms to find equivalent frequency sinusoidal-shaped waveforms, the characteristic frequency ( $f_{c}$) of our square-shaped waveforms may be more simply approximated as follows:

$$f_{c}=\frac{1}{ON+\mathrm{OFF}+ON+\mathrm{OFF}}.$$ (1)

Per Eq. (1), an alternate H-FIRE pulsing paradigm to match the 100 kHz B-SWE waveform could have been employed with a near equivalent characteristic frequency by utilizing a 3-2-3 μs waveform rather than the 2-3-2 μs waveform. However, as a goal of this study was to maximize BBB disruption while minimizing ablation, previous literature has indicated that ablation may be minimized by reducing the duration of the pulse width, and BBB disruption may be maximized by increasing the duration of the pulse delay.^12^

### B. Validation of B-SWE and H-FIRE protocols on BBB disruption and ablation

Following the specified pulsed electric field (PEF) treatment, either B-SWE or H-FIRE, a comprehensive investigation of BBB disruption and tissue ablation following electroporation was conducted through a comparative analysis of two distinct outcome measures: (1) BBB disruption measured using either T1-weighted contrast-enhanced MRI with gadopentetate dimeglumine (Gd-DTPA) enhancement 1 h after PEF treatment or Evans blue dye (EBD) uptake measured on gross pathology, and (2) ablation volume assessed 24 h post PEF treatment via T2-weighted MRI.

**FIG. 2.**
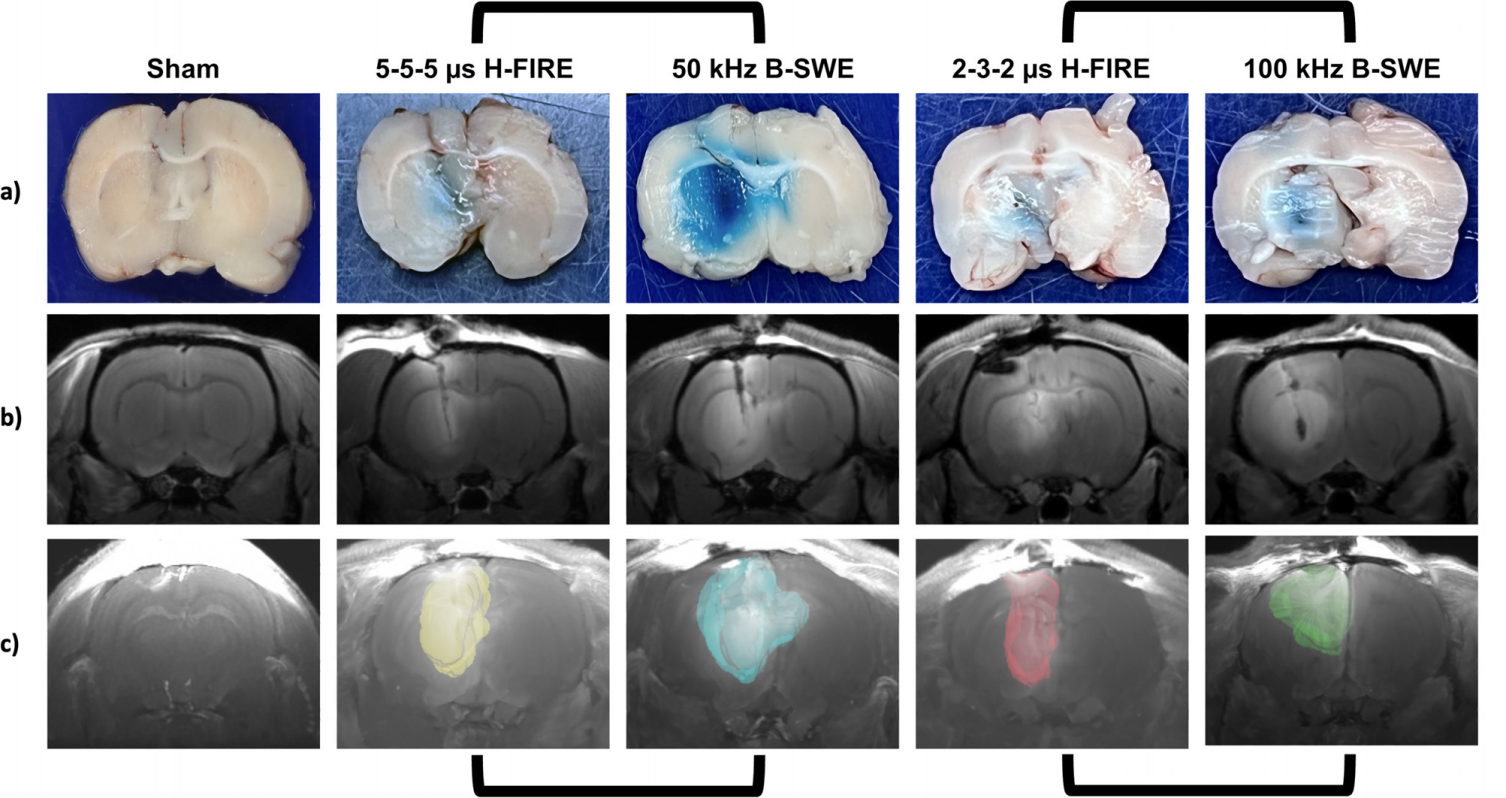
A visualization of BBB disruption 1 h post-treatment as noted by the diffusion of normally impermeant (a) Evans blue dye (EBD) and (b) gadopentetate dimeglumine (Gd-DTPA) into the striatum. Volumetric measurements of EBD were taken by measuring tissue sections while (c) contrast-enhanced regions of Gd-DTPA uptake on T1W MRI scans were contoured and quantified. Both EBD and Gd-DTPA volumetric measurements demonstrate greater BBB disruption from an equivalent B-SWE protocol. All tissue sections and scan slices depict planes of BBB disruption along or orthogonal to the electrode insertion tip. Paired protocols are matched via brackets.

**FIG. 3.**
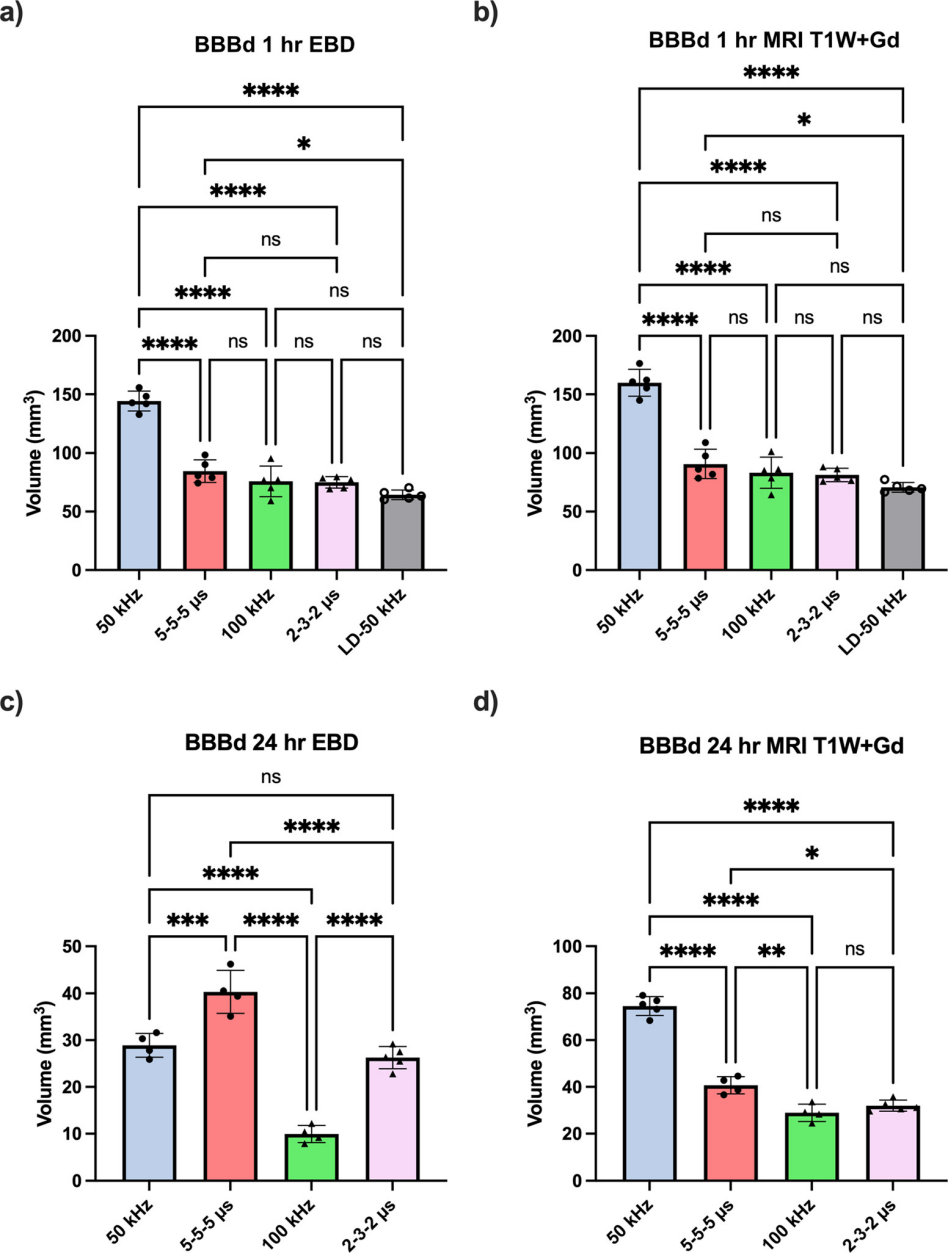
Blood–brain barrier disruption (BBBd) volumes using Evans blue dye (EBD) and gadopentetate dimeglumine (Gd-DTPA) volumetric measurements. BBBd 1 h after an equivalent burst sine wave electroporation (B-SWE) protocol reveals a significant increase in the 50 kHz B-SWE compared to its equivalent traditional 5-5-5*μ*s square-shaped waveform. EBD, a molecule ∼ 70× larger than Gd-DTPA demonstrates slightly less uptake at the 1-h time point post-treatment compared to Gd-DTPA uptake (a) and (b); however, a substantial difference is observed at the 24-h time point (c) and (d). Matched waveforms are designated by matched data point symbols. ^*^ denotes a p-value < 0.05, ^**^ denotes a p-value < 0.01, ^***^ denotes a p-value < 0.001, and ^****^ denotes a p-value < 0.0001.

Gd-EBD solutions were administered 1 h prior to euthanasia to allow ample time for the solution to circulate. One hour following the selected PEF treatment, anesthetized rats were humanely euthanized by IP pentobarbital (0.5 ml) overdose (Fatal Plus, Vortech Pharm, Dearborn, MI, USA) to be evaluated for BBB disruption. Rats were subject to T1-W MRI for quantification of Gd-DTPA enhancement, and then a craniectomy was performed immediately after to quantify uptake of EBD into the brain parenchyma. Both T1-W MRI and gross pathology measurements revealed a consistent pattern wherein B-SWE protocols exhibited at least equal or larger volumes of BBB disruption compared to their equivalent H-FIRE waveforms at the 1 h time point upon gross examination (Fig. 2). A robust enhancement of BBB disruption with both Gd-DTPA and EBD was specifically observed in the 50 kHz B-SWE protocol, highlighting its efficacy in inducing the greatest amount of BBB disruption among all the investigated groups (Fig. 3).

**FIG. 4.**
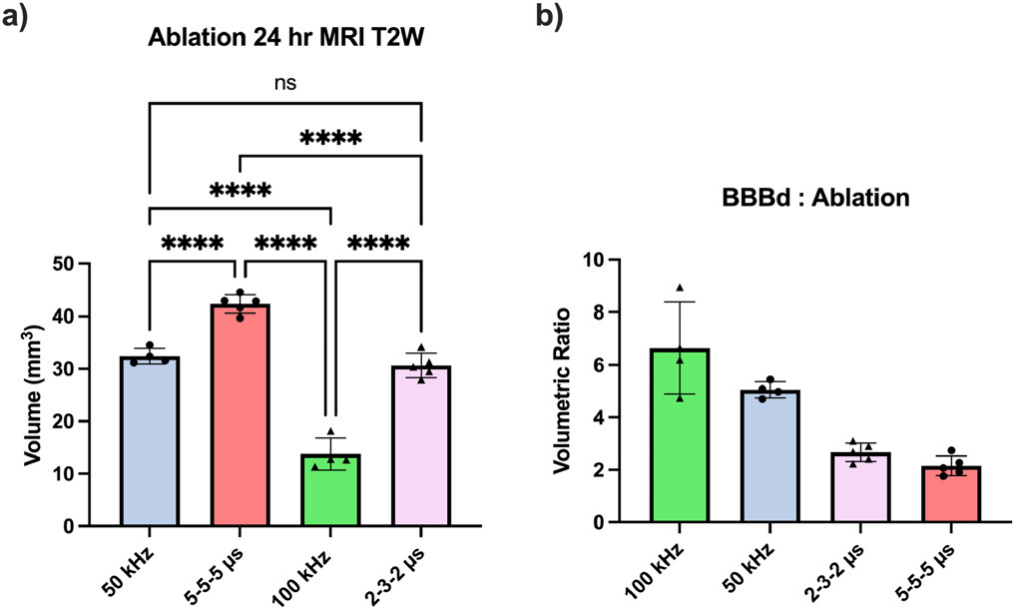
(a) Ablation volumes 24 h post-electroporation (T2-W MRI). H-FIRE waveforms consistently induce larger volumes of tissue ablation than their equivalent B-SWE waveform. (b) Burst sine wave electroporation (B-SWE) protocols demonstrate greater blood–brain barrier disruption (BBBd) to ablation ratios than traditional square-shaped wave H-FIRE, with a decreasing ratio as characteristic frequency decreases. Matched waveforms are indicated by matched individual data point symbols. ^*^ denotes a p-value < 0.05, ^**^ denotes a p-value < 0.01, ^***^ denotes a p-value < 0.001, and ^****^ denotes a p-value < 0.0001.

For the evaluation of tissue ablation, rats were euthanized 24 h post-treatment. This specified waiting period is required as non-thermal tissue ablation induced by electroporation-based technologies may take up to 24 h to fully evolve.^13^ Similarly to BBB disruption evaluation protocols, Gd-EBD solution was administered 1 h pre-euthanasia (23 h post-treatment) to allow for the complete circulation of the agents. Contrary to the observations for BBB disruption, H-FIRE waveforms consistently produced greater tissue ablation than their equivalent B-SWE counterparts [Fig. 4(a), p < 0.0001 5-5-5 *μ*s vs 50 kHz and 2-3-2 *μ*s vs 100 kHz]. Additionally, lower frequencies within both square and sinusoidal groups create larger volumes of tissue ablation [Fig. 4(a), p < 0.0001 5-5-5 vs 2-3-2 *μ*s and 50 vs 100 kHz].

It is thereby noted that B-SWE protocols are capable of inducing relatively large volumes of BBB disruption in comparison to the controlled volumes of tissue ablation [Fig. 4(b)], whereas H-FIRE protocols may induce greater volumes of tissue ablation if desired. From these results, clinicians may make the choice between an H-FIRE protocol for a large BBB disruption and moderate ablation in the context of treating large and dispersing tumors, or a B-SWE protocol for a large BBB disruption while inducing only minimal ablation where tumors may be situated near sensitive structures. Other applications have indicated that PEFs may be capable of relieving the hypoxic tumor environment which has been detrimental to radiotherapy success.^14^ Additionally, the potential application of electroporation in non-cancerous neurological conditions, such as Alzheimer's or Parkinson's disease, where BBB disruption is essential for effective drug uptake, is also considered.

### C. Effects of waveform selection on neuromuscular contractions

**FIG. 5.**
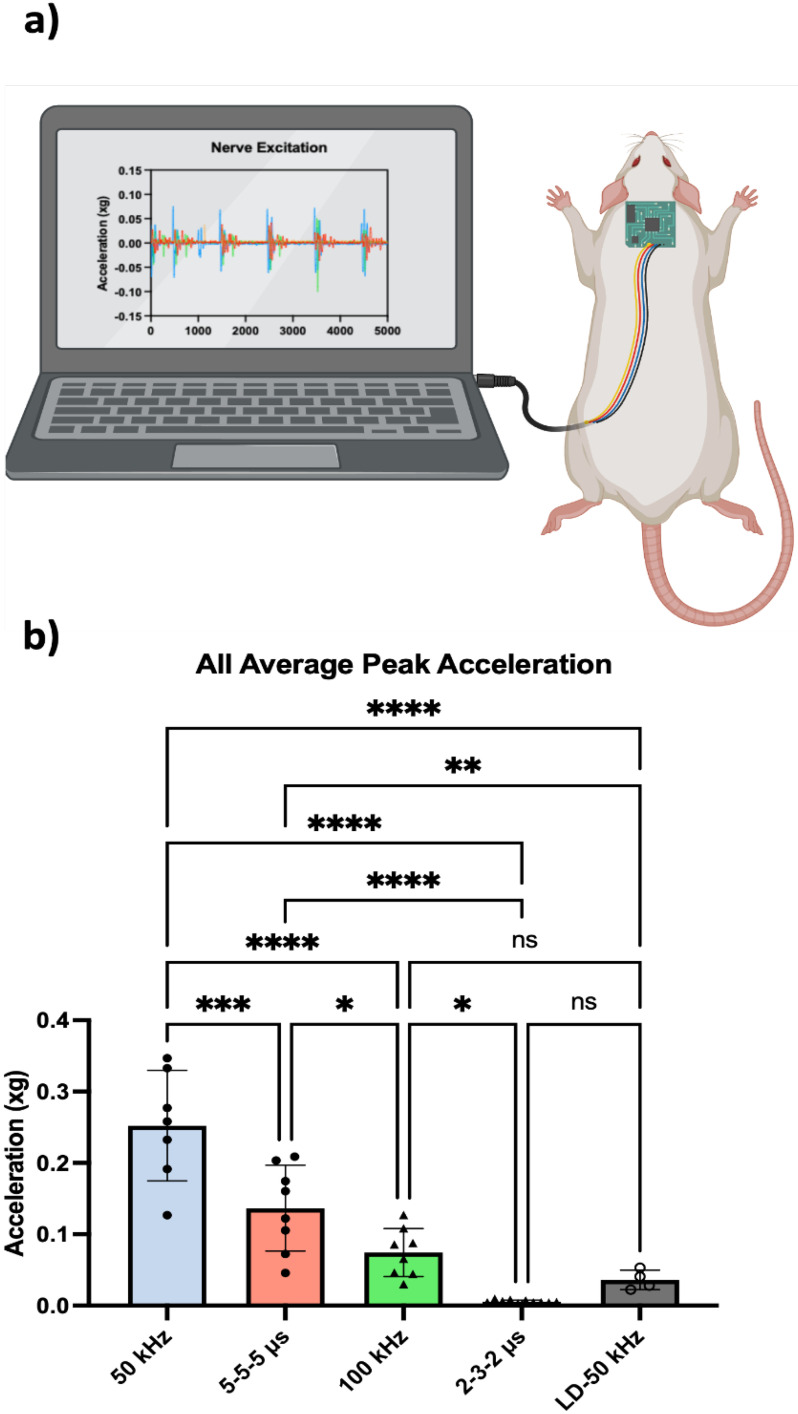
Intra-treatment acceleration data analyzed through (a) accelerometry measurements acquired from a gyroscope sensor positioned on the base of the posterior skull and connected to a microcontroller to record muscle twitching at ∼300 Hz. (b) Peak acceleration values measured from the vertical (z-axis) component of the accelerometer were plotted and compared between waveforms. Peak acceleration values from each treatment were plotted indicating a decrease in muscular stimulation at higher frequencies, but greater muscular stimulation from B-SWE protocols in comparison to the equivalent H-FIRE protocol. A lower dose 50 kHz sine wave protocol (LD-50 kHz) was implemented to achieve less neuromuscular contractions than the equivalent frequency 5-5-5 H-FIRE group. ^*^ denotes a p-value < 0.05, ^**^ denotes a p-value < 0.01, ^***^ denotes a p-value < 0.001, and ^****^ denotes a p-value < 0.0001.

As neuromuscular contractions are occasionally a secondary effect of PEF-based therapies, we sought out to elucidate the effects of paired B-SWE and H-FIRE waveforms on this effect. The graphical depiction of peak acceleration values in each treatment, as illustrated in Fig. 5, revealed noteworthy trends. Specifically, a clear reduction in muscular stimulation was evident at higher frequencies. However, despite this trend, B-SWE protocols displayed heightened muscular stimulation compared to their equivalent H-FIRE counterparts [Fig. 5(b), p < 0.001 5-5-5 *μ*s vs 50 kHz and p < 0.01 2-3-2 *μ*s vs 100 kHz]. Expectedly, lower frequencies within both square and sinusoidal groups results in greater peak acceleration [Fig. 5(b), p < 0.0001 5-5-5 vs 2-3-2 *μ*s and 50 vs 100 kHz]. Given the gap present in extent of BBB disruption between the 50 kHz and 5-5-5 *μs* waveform pair, a lower dose 50 kHz B-SWE protocol (LD-50 kHz) was implemented to induce fewer neuromuscular contractions than other protocol groups, while still maintaining a level of BBB disruption comparable to that of higher dose counterparts [Figs. 3(a) and 3(b)].

Within the LD-50 kHz group, a significant reduction in neuromuscular contractions was observed compared to its equivalent frequency H-FIRE waveform, albeit with a lower applied potential. A comparable degree of BBB disruption was measured between the two groups. Despite minor differences in these volumes, a degree of statistical significance was noted. Due to the strength of the statistical difference between the peak muscle contractions measured in the LD-50 kHz B-SWE and 5-5-5 *μs* H-FIRE being more statistically different ( p<0.01) than the difference noted in BBB disruption ( p<0.05), one may expect that the applied potential of the LD-50 kHz may be raised just enough to induce an equivalent amount of BBB disruption without compromising the reduction noted in muscle contraction. Given that the original 50 kHz (peak voltage of 680 V) B-SWE waveform exhibited less ablation than its equivalent 5-5-5 H-FIRE waveform, it is reasonable to anticipate that the lower applied voltage magnitude of the LD-50 kHz B-SWE waveform would lead to a further reduction in ablation.

The 2-3-2 *μs* H-FIRE waveform exhibited minimal acceleration, as evidenced by negligible accelerometer recordings [Fig. 5(b)] as well as a visual absence of twitching during treatment delivery. A plausible explanation for this observation could be linked to the identified characteristic frequencies. In Fig. 1(d), the 2-3-2 *μs* waveform displays two prominent peaks exceeding 50% of the normalized values, situated at approximately 100 and 300 kHz. It is conceivable that the heightened intensity of these higher frequency components contributes to a more effective mitigation of muscle contractions. While the normalized peak maximum value [magnitude = 1; Figs. 1(c)–1(f)] serves as an indicator of the predominant behavior of the waveform, we recommend considering the calculation of a modified weighted characteristic frequency in future studies. This modification could provide a more comprehensive understanding of the waveform's behavior, ensuring a thorough capture of its dynamics. To fully capture the behavior of the waveform, the authors propose the following weighted characteristic frequency term $f_{\text{weighted}}$:

$$f_{\text{weighted}}=\frac{\sqrt{{\left(w_{1}*f_{1}\right)}^{2}+{\left(w_{2}*f_{2}\right)}^{2}}\ldots }{w_{1}+w_{2}\ldots },$$ (2)

where $w_{x}$ represents the specific normalized weighting factor and $f_{x}$ represents the frequency at the respective peak. Comparison of the weighted frequencies are reported in Table I.

**TABLE I.** Equivalent frequencies.

Waveform	Frequency	FFT max frequency	Weighted frequency
B-SWE	50 kHz	50.000 kHz	50.000 kHz
H-FIRE	5-5-5 *μs*	49.998 kHz	60.561 kHz
B-SWE	100 kHz	100.000 kHz	100.000 kHz
H-FIRE	2-3-2 *μs*	99.990 kHz	126.658 kHz

The dataset presented offers essential insights into the dynamic response of muscle activity during electroporation procedures, demonstrating that lower applied fields through B-SWE protocols may be able to relieve severe muscular contractions, while maintaining comparable volumes of BBB disruption.

### D. Electric field thresholds

Establishing the electric field threshold (EFT) for electroporation-based technologies is a critical aspect of the treatment planning process. This step is essential to understand the borders of the predicted ablation zone to maximize the preservation of healthy tissues. Achieving this balance contributes to enhanced patient outcomes. In the context of BBB disruption, it is essential to understand the implications of pulsing parameters to ensure diffuse BBB disruption.

**FIG. 6.**
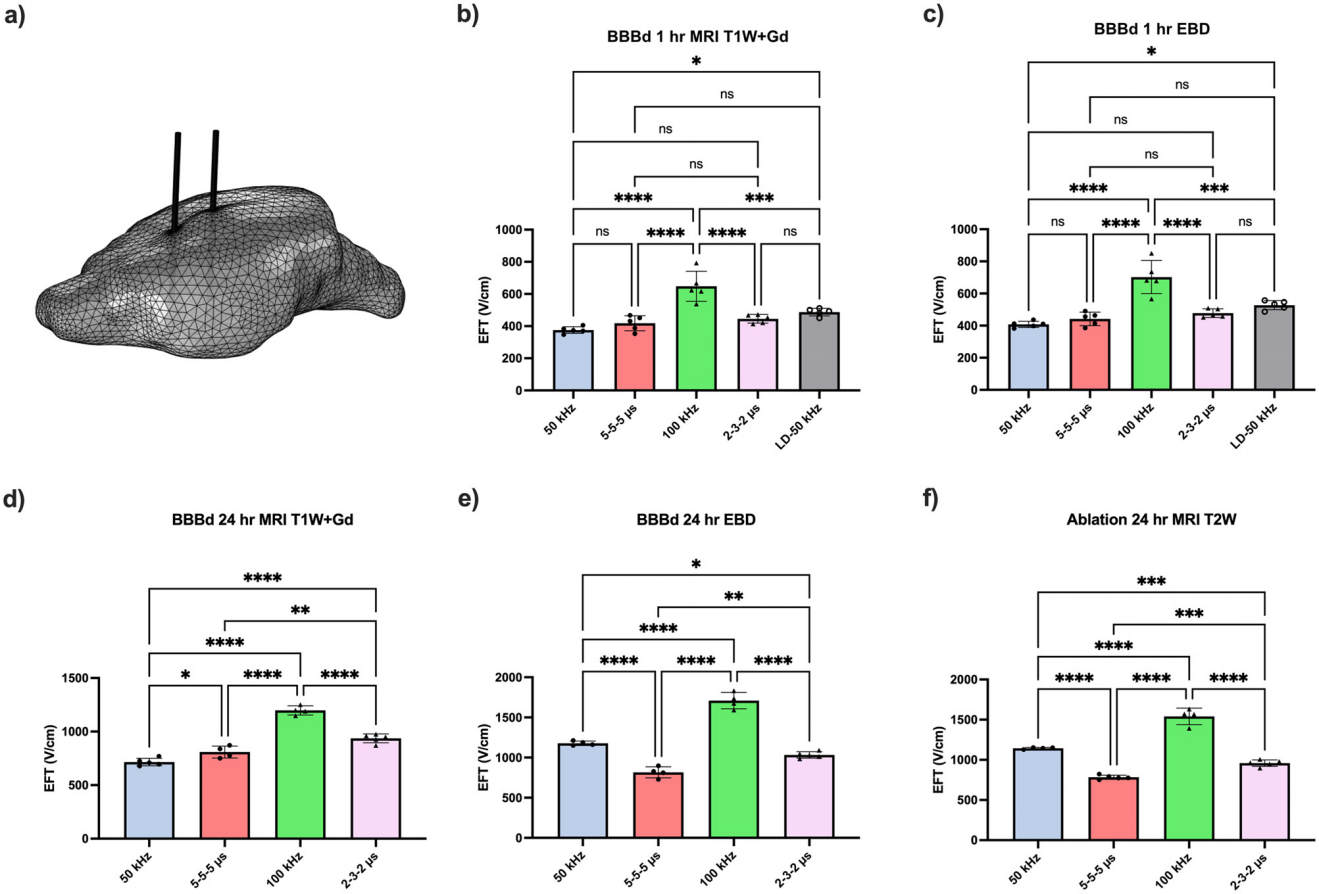
(a) Representation of the rat brain mesh built for the numerical model, constructed and executed to simulate experimental conditions. Electric field thresholds (EFT) were extracted for various waveform types and different measurement quantifications (BBB disruption: either T1-weighted contrast-enhanced MRI or EBD gross pathology; ablation: T2-weighted MRI). The extracted EFTs are presented for BBB disruption time points at (b) and (c) 1 h post-treatment, (d) and (e) 24 h post-treatment, and (f) ablation EFTs were also quantified. Paired waveforms are indicated by matched individual data point symbols. ^*^ denotes a p-value < 0.05, ^**^ denotes a p-value < 0.01, ^***^ denotes a p-value < 0.001, and ^****^ denotes a p-value < 0.0001.

Using finite element modeling to recreate the experimental conditions and match the measured outcomes for BBB disruption and ablation, respectively, to the contour in the numerically generated field distribution, the EFTs were extracted and are shown in Fig. 6.

### E. Thermal considerations

Numerical models were used to estimate the temperature dynamics during treatment (supplementary material Fig. 2). With initial conditions set to a physiological brain temperature of 37 °C, our simulations projected maximum temperatures after 200 bursts as follows: 39.07 °C for 5-5-5 H-FIRE, 40.37 °C for 50 kHz B-SWE, 38.301 °C for LD-50 kHz B-SWE, 39.08 °C for 2-3-2 H-FIRE, and 40.39 °C for 100 kHz B-SWE.

These estimations closely mirror temperatures reported in the literature for irreversible electroporation (IRE) in the brain.^15^ Despite the absence of direct temperature measurements during treatment, our gross examination of the excised tissues did not reveal any signs of thermal damage, such as excessive coagulation or hemorrhaging.

### F. Blood–brain barrier disruption only protocols

The exploration of B-SWE as a method to induce diffuse BBB disruption without causing ablation is particularly intriguing. Examining the EFTs depicted in Fig. 6, it becomes evident that B-SWE protocols require higher EFTs for inducing ablation. This implies that a greater field strength needs to be exceeded to trigger ablation in comparison to conventional square wave treatments. The reported EFT values are derived from both root mean square voltages ( $V_{\mathrm{RMS}}$) and peak voltages ( $V_{pk}$) for H-FIRE and B-SWE, respectively. Notably, the EFTs for 50 kHz B-SWE, aimed at inducing BBB disruption, fall within the range of equivalent H-FIRE 5-5-5 μs EFTs, thus, B-SWE EFTs at or below $EFT_{\mathrm{VRMS}}$ result in BBB disruption volumes equal to or greater than those achieved with equivalent H-FIRE waveforms. This is attributed to $V_{pk}>V_{\mathrm{RMS}}$, enabling a diffuse field distribution.

**FIG. 7.**
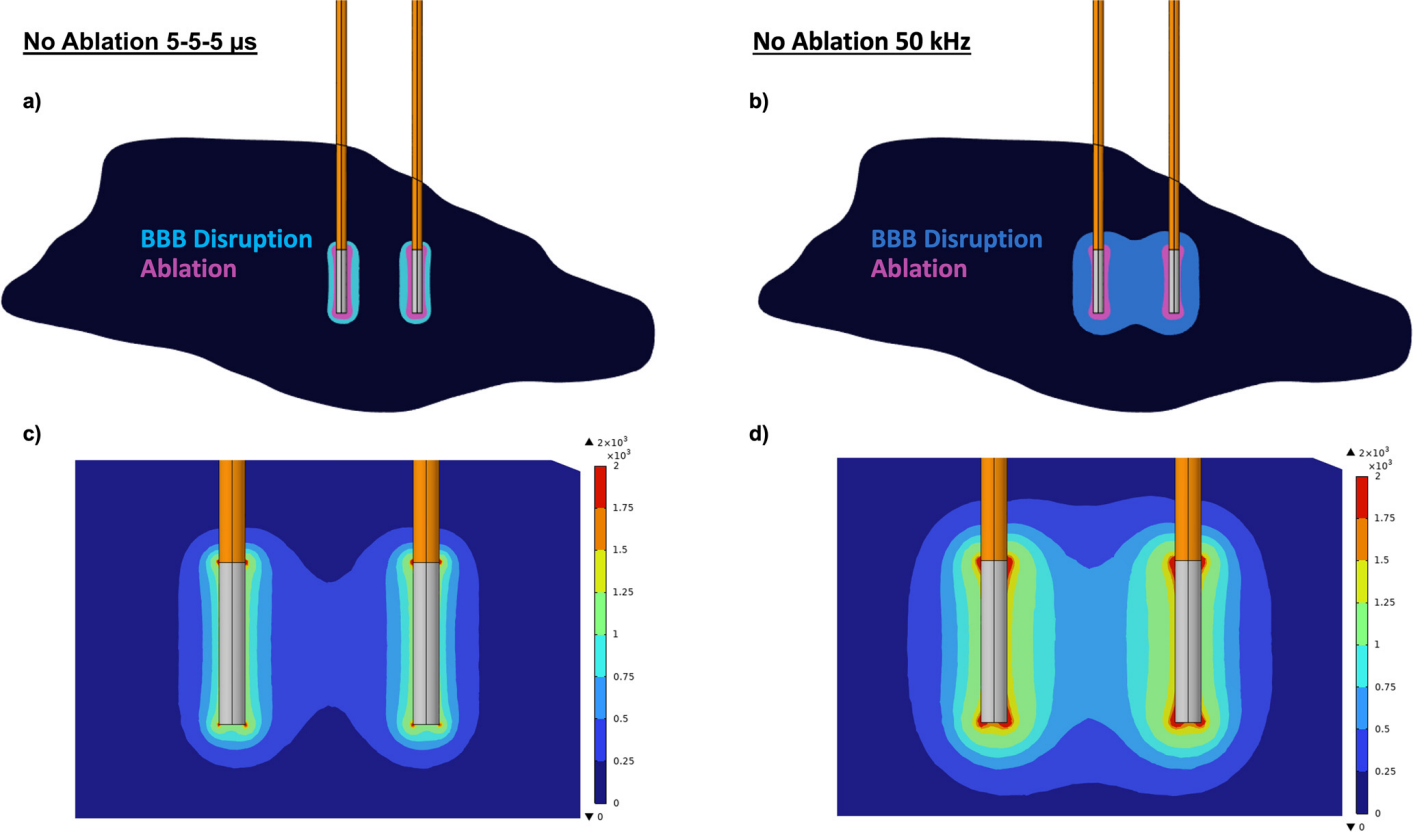
The EFTs extracted from the 50 kHz B-SWE and 5-5-5 H-FIRE for ablation and 1 h BBB disruption were utilized to sweep voltages and ascertain the maximum voltage that would generate ablation lesions smaller than the volume of the active probes (1.64 mm^3^). The resulting voltages to satisfy this condition were found to be 170 V for 5-5-5 H-FIRE and a peak voltage of 410 V for the 50 kHz B-SWE. (a) and (b) Visual representation of the estimated field-induced phenomenon for a designed protocol aimed at minimizing ablation. (c) and (d) The resulting electric field distribution illustrates that the 50 kHz sine wave effectively generates a broader distribution of lower strength fields, contributing to blood–brain barrier (BBB) disruption.

One can optimize the applied potential to create a field distribution slightly below the ablation EFT but substantially above the EFT for BBB disruption ( $EFT_{Ab}>EF_{\text{Applied}}>EFT_{\mathrm{BBBd}}$). By selecting the highest potential meeting these criteria, it is noteworthy that the electric field peak in B-SWE protocols significantly surpasses that in traditional square-shaped H-FIRE. This discrepancy in the EFTs between ablation and BBB disruption contributes to the possibility of achieving minimal ablation with a relatively extensive BBB disruption. The finite element modeling software, COMSOL Multiphysics (Comsol, Stockholm, Sweden), was used to simulate a prospective treatment condition and plot the field distribution that would induce an equivalent ablation only confined to the electrode surface while examining the resulting diffuseness of BBB disruption-inducing fields (Fig. 7).

### G. Blood–brain barrier repair kinetics

**FIG. 8.**
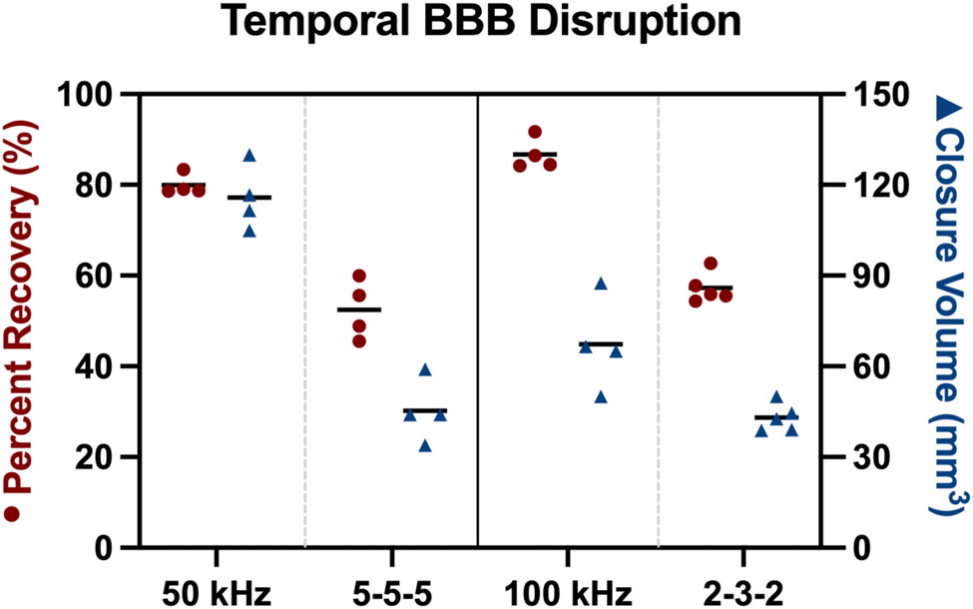
Blood–brain barrier (BBB) disruption kinetics were evaluated based on the changes in uptake volumes of Evans blue dye at the 1- and 24-h post-treatment time points. Percent recovery (red circles) indicate a percent volume change between the 1- and 24-h post-treatment time points for BBB disruption. The closure volume (blue triangles) indicates the volume of the previously disrupted BBB (1 h) that undergoes closure (24 h). For larger molecules, such as Evans blue dye, burst sine wave electroporation-induced BBB disruption has quicker repair kinetics than traditional square-shaped wave H-FIRE.

The observed differences in repair kinetics between B-SWE and traditional square-shaped wave H-FIRE in the context of EBD uptake provide insight on the temporal dynamics of BBB closure (Fig. 8). Notably, B-SWE protocols demonstrated a substantial change in the uptake kinetics of EBD, with an 80%–87% change in molecule uptake ability, while traditional H-FIRE protocols exhibited a comparatively modest BBB closure in the range of 52%–57%. Although our study was not explicitly designed to investigate the temporal nature of these waveforms, the data suggest that within the initial 24-h period following the PEF therapy, B-SWE-induced BBB disruption experiences faster repair kinetics.

The clinical implications of this finding are significant, particularly in the context of therapeutic applications that rely on transient BBB disruption to facilitate the delivery of otherwise impermeable drugs. However, a delicate balance must be struck, as prolonged disruption of the BBB may create a vulnerable window, potentially allowing the entry of toxins or circulating pathogens into the central nervous system. Achieving the optimal balance between maximizing the therapeutic advantage of BBB disruption and ensuring timely closure to protect against potential harmful pathogens becomes paramount. Of note, smaller molecules like Gd-DTPA (approximately 1 kDa), roughly 70 times smaller than EBD, exhibit expected BBB penetration trends 24 h post-treatment regardless of waveform type. Conversely, large molecules, such as EBD (approximately 69 kDa), do not demonstrate this penetration pattern 24 h post-treatment for B-SWE, although it remains viable for traditional H-FIRE. Therefore, prolonged exposure to smaller molecules around 1 kDa may persist for a duration comparable to that observed in traditional H-FIRE protocols, as demonstrated by Lorenzo *et al.*^5^ This temporal discrepancy suggests that larger defects initially induced during B-SWE, facilitating the penetration of large molecules like EBD, may contract or close by the 24-h time point. This observation implies that the B-SWE protocol potentially generates more diffuse regions of BBB disruption characterized by smaller sized defects. This finding is further supported by the 24-h EBD results [Fig. 3(d)], highlighting a contrast between the potentially more diffuse but smaller defects induced by B-SWE compared to the less diffuse but larger defects created by traditional H-FIRE.

Future research efforts may delve deeper into understanding the intricate interplay between the duration of BBB disruption and the associated risks and benefits, ultimately guiding the development of precise and targeted drug delivery.

The observed trends in BBB disruption and tissue ablation provide valuable insights into the differential effects of B-SWE and H-FIRE waveforms on brain tissue integrity. The sustained larger volumes of BBB disruption induced by B-SWE suggest its potential as a targeted and efficient modality for transiently permeabilizing the BBB. However, the consistently larger tissue ablation volumes associated with H-FIRE waveforms at the 24-h time point emphasize their capacity for inducing more substantial and prolonged effects on tissue structure.

## III. CONCLUSIONS

The preference for sharp rise and fall times inherent in square-shaped waves is attributed to the minimal transient changes in the electric field, ensuring that the electric field remains at or above the desired threshold throughout the pulse duration. The overall goal of this work was to introduce diffuse regions of BBB disruption to enhance drug delivery for targeting infiltrating glioblastoma cells that have migrated beyond the bulk tumor volume (commonly associated with the difficulty in full targeting of the disease) while maintaining precise control over the extent of high-strength ablation fields confining the ablation regions to the tumor volumes, minimizing cell death to healthy neurological tissues. This nuanced exploration reveals the intricate interplay between electroporation parameters, neuromuscular response, tissue ablation induction, and BBB disruption. Such insights are invaluable for refining the design and application of electroporation protocols across diverse biomedical contexts. The significant advantage of this work lies in its contribution to tailoring treatment protocols based on specific therapeutic goals. Clinicians can choose between H-FIRE protocols for large BBB disruption and moderate ablation in treating expansive tumors or opt for B-SWE protocols for substantial BBB disruption with minimal to no ablation in sensitive areas. The application of electroporation in post-surgical cerebral regions to enhance therapeutic delivery without compromising healthy tissue to ablation strength fields is highlighted.

## IV. METHODS

### A. Determining equivalent sinusoidal and square-shaped waveforms

In our approach to waveform matching, we prioritize voltage-based equivalence due to its fundamental role in governing the phenomenon of electroporation. The transmembrane potential ( $\Psi_{m}$) serves as a key determinant in this process, governing the response of cell membranes to external electric fields. As detailed in Eq. (3), $\Psi_{m}$ is influenced by factors such as the electric field ( $\vec{E}$), cell radius (*R_c_*), and shape factor (*f_s_*), the latter primarily influenced by the electrical properties of the surrounding medium. Hence, by mitigating factors specific to the pulsing medium rather than the applied waveform, the electric field term is dominant. Thus, voltage directly influences the strength of the electric field, which is crucial for inducing membrane permeabilization.

$$\Psi_{m}=f_{s}*\vec{E}*R_{c}*\;\text{cos}\;\varphi *(1-e^{-\frac{t}{\tau }}).$$ (3)

The characteristic frequency of the square-shaped waveform, representing the frequency at which the square-shaped waveform most closely resembles a sinusoidal-shaped waveform, was determined by Eq. (1). The characteristic frequency was then validated using fast Fourier transform (FFT), which provided a spectral representation of the square-shaped waveform. The frequency component with the highest amplitude in the spectral representation corresponded to the characteristic frequency, thereby verifying the value obtained from Eq. (1).

Waveform equivalence based on frequency and applied voltage components were thereby verified as follows:

$$\begin{array}{c} \int_{0}^{{\text{BurstDuration}}_{\text{sq}}}|V_{\text{rms}}(t)|=\int_{0}^{{\text{EnergizedTime}}_{\text{sw}}}|V_{pk}\times \text{sin}(2\cdot \pi \cdot t\cdot \omega )|, \end{array}$$ (4)

where ${\text{BurstDuration}}_{sq}$ refers to the total amount of time it takes to deliver a full H-FIRE burst in which the pulsing cycle (which includes a positive pulse, intra-phase delay, negative pulse, and inter-cycle delay) is repeated until the desired energized time is reached. ${\text{EnergizedTime}}_{sw}$ is the total energized time of the sine wave and ω refers to the frequency of the sine wave. $V_{\text{rms}}$ is the applied voltage of the square-shaped wave which provides the equivalent area under the applied voltage potential curve as a matched sinusoidal waveform,

$$V_{\text{rms}}=\frac{V_{pk}}{\sqrt{2}},$$ (5)

where $V_{pk}$ is the peak voltage of the sinusoidal waveform.

### B. *In vivo* treatment delivery

#### 1. Assurances and surgical procedures

The study was conducted adhering to the principles delineated in the Guide for the Care and Use of Laboratory Animals and received approval from the Institutional Animal Care and Use Committee (IACUC #  23-024). Adult male Fischer rats, with a weight range of 150–200 g at the time of treatment, were employed in the research. Following a one-week acclimation period, the animals underwent pre-medication with a subcutaneous injection of buprenorphine (Ethiqa XR; Fidelis Pharmaceuticals, North Brunswick, NJ) at a dosage of 0.65 mg/kg.

Anesthesia was induced using a 3%–4% mixture of isoflurane and 95% oxygen, and maintenance was achieved with a 2%–3.5% mixture of isoflurane and 95% oxygen delivered through a nosecone. The dorsal region of the head, spanning from the intercanthal area to the cranial cervical region, was clipped and prepared for aseptic surgery. Subsequently, the animals were positioned in a small animal stereotactic headframe (Model 1350M; David Kopf Instruments, Tujunga, CA, USA) and underwent a unilateral rostrotentorial surgical approach to the skull. Using a high-speed electric drill (Dremel 3000 Series; Mount Prospect, IL, USA) with a 2.4 mm diameter round burr, a 4 × 2.5 mm^2^ rectangular parietal craniectomy defect was then created in the skull of each rodent.

#### 2. Delivery of pulsed electric fields

On the day of treatment, the electrodes were inserted into the brain using the micromanipulator arm of the stereotactic frame, guided by stereotactic coordinates referencing the location of the rostral electrode (bregma 4 mm caudal, 3.5 mm lateral, at a depth of -4 mm relative to the surface of the dura). The treatment involved delivering 200 bursts at a rate of 1 burst per second across two blunt-tipped stainless steel electrodes (26G; exposure = 2.5 mm; spacing = 3.0 mm). The applied voltage was set to either a peak voltage of 680 V for the sinusoidal waveform or a root mean square voltage 480 V for the square waveform. To explore the potential of minimizing muscle contractions while maintaining comparable blood–brain barrier (BBB) disruption volumes as observed in the 5-5-5 H-FIRE group, an additional low-dose 50 kHz (LD-50 kHz) B-SWE group was introduced. As this additional group was conducted following initial results from the above-mentioned groups, EFTs were calculated for the 50 kHz group (peak voltage of 680 V) using the methods outlined in Sec. IV D. Subsequently, forward simulations were conducted to estimate the applied voltage required to induce a BBB disruption volume akin to that of the 480 V 5-5-5 H-FIRE waveform. Therefore, a peak voltage of 460 V was used.

The following experimental conditions were investigated:

• 50 kHz B-SWE at 680 *V_pk_* at 1 h (n = 5)
• 50 kHz B-SWE at 680 *V_pk_* at 24 h (n = 5)
• 100 kHz B-SWE at 680 *V_pk_* at 1 h (n = 5)
• 100 kHz B-SWE at 680 *V_pk_* at 24 h (n = 5)
• 5-5-5 μs H-FIRE at 480 V at 1 h (n = 5)
• 5-5-5 μs H-FIRE at 480 V at 24 h (n = 5)
• 2-3-2 μs H-FIRE at 480 V at 1 h (n = 5)
• 2-3-2 μs H-FIRE at 480 V at 24 h (n = 5)
• LD-50 kHz B-SWE at 460 *V_pk_* at 1 h (n = 5)

(Total sample: N = 45)

#### 3. Procedures for measuring BBB disruption and ablation volumes

In order to assess the permeability of the BBB, an intraperitoneal injection of a solution containing gadopentetate dimeglumine (Gd-DTPA) at a concentration of 0.1 mmol/kg and 2.5% Evans blue dye (EBD) at a dose of 75 mg/kg was administered to anesthetized rodents. It is noteworthy that Gd-DTPA and EBD are incapable of permeating the intact BBB, and hence, their presence in the brain parenchyma would imply a disruption of the BBB following pulsed electric fields (PEF) treatment. The Gd-EBD solution was administered 1 h prior to euthanasia. Thus, in the 1 h group, Gd-EBD was injected 5 min prior to treatment, while in the 24-h group, Gd-EBD was injected 23 h post PEF treatment. This allows sufficient time for Gd-EBD to circulate systemically and permeate the regions with a disrupted BBB. This time point was chosen based on prior work by Lorenzo *et al.*, who determined that the maximum BBB disruption is noted 1 h post PEF treatment.^5^ The rats were euthanized using an intraperitoneal pentobarbital overdose at either 1 h or 24 h post-treatment, depending on whether or not they would be evaluated for the extent of BBB disruption or ablation, respectively.

Brain MRI images were obtained immediately on a 9.4 T scanner (Bruker Biospec 9.4T, Bruker Billerica, MA, USA). T1W 2D FLASH images were obtained using the following acquisition parameters: TR/TE: 230/3.3 ms, FA: 70°, resolution = 86 × 86 × 500 *μ*m^3^. The quantification of the BBB disruption volumes and ablation volumes was based on the contrast-enhanced regions in T1-weighted scans and hyperintense regions in T2-weighted scans respectively.

Following the MRI imaging, the rodents' brains were promptly extracted and fixed in a 10% neutral buffered formalin solution. After a 48-h incubation period, the brains were sliced in the transverse plane at 2 mm intervals using an adult rodent matrix slicer (Ted Pella Inc., Redding, CA, USA). Each transverse slice was photographed. The positions of the transverse sections where the first and last visible sections of Evans blue dye (EBD) were detected, representing the anterior/rostral and posterior/caudal limits of the z-plane of blood–brain barrier (BBB) disruption, were recorded.

The transverse brain sections containing EBD within these limits were further sub-sectioned serially at a thickness of 10 μm and intervals of 200 μm using a microtome, and then mounted on microscope slides. Digital photomicrographs of the intraparenchymal EBD were captured from all intervening transverse sections using a charge-coupled device camera equipped with a fixed aperture. The volume of EBD was quantified for each rat's transverse image stack using a Cavalieri estimator on a commercial image analysis system (Stereo Investigator; MBF Bioscience, Williston, VT, USA).

### C. Acceleration

Accelerometry measurements were obtained using a 3-axis gyroscope sensor (ADXL337, Analog Devices, Norwood, MA) positioned at the base of the posterior skull behind the location of the defect. The sensor was connected to a microcontroller recording positioning data at a rate of ∼300 Hz. Data points were transferred into MATLAB 2021b for analysis. The magnitude of peak acceleration values extracted from the vertical (z-axis) component of the accelerometer was plotted and compared against equivalent waveforms.

### D. Evaluation of BBB disruption and ablation thresholds *in vivo*

The experimental conditions were recreated using COMSOL Multiphysics (Comsol, Stockholm, Sweden), with methods similar to those presented in Campelo *et al.*^10,16^ (supplementary material Table I). To optimize computational efficiency while maintaining accuracy during burst periods, a dynamic time-stepping approach was implemented. The sine wave was broken down into 9 evenly spaced points along the single cycle of the specified sine wave, where the intra-burst time step sizes for the 100 and 50 kHz B-SWE waves were 1.25 and 2.5 μs respectively [Eq. (6)]. Within each burst, these small time steps were used to capture essential dynamics contributing to the Joule heating generation. However, to mitigate computational resource constraints, the time step was momentarily increased to align the solver with the nearest full second, matching the burst repetition frequency of 1 s. This adjustment occurs immediately after burst completion, ensuring that the 100 *μ*s energized waveform repeats once every second. Square waveforms were modeled as previously established in the literature with a duty cycle approach.^17^

$$\text{stepsize}=\frac{1}{\text{frequency}}*\frac{1}{8}.$$ (6)

A 3D reconstruction of a rat brain MRI was smoothed and imported into the finite element modeling software for an accurate representation of electric field distributions [Fig. 6(a)]. Individual dynamic conductivity curves were generated for each protocol by fitting voltage vs current data generated computationally to match experimentally acquired voltage ramp data (supplementary material Fig. 1).

Dynamic conductivity curves for each protocol were constructed using techniques akin to those described by Campelo *et al.*,^10^ where computationally generated voltage vs current data were fit to align with experimentally acquired voltage ramp data. In essence, a pre-treatment voltage ramp with increments ranging from 25 V to the treatment voltage was applied and both current and voltage readings were recorded. The parameters of the computational model's conductivity curve were adjusted iteratively until the computational model output exhibited the same voltage–current combinations observed in the experimental data. A nonlinear exponential fit was used to determine the acceptability of the fit between experimental and numerical datasets. $R^{2}$ values above 0.95 and a p > 0.05 indicating that there is no significant difference between each datasets' best fit curves were used as confirmation metrics.

For each waveform, the initial ( $\sigma_{0}$) and maximum ( $\sigma_{f}$) conductivity values were adopted from previously reported frequency-dependent conductivity data from Gabriel *et al.*^18^ Their experimental findings provide estimated tissue conductivity values under specific applied frequency voltages. Thus, $\sigma_{0}$, was taken to be the conductivity under our applied waveform's characteristic frequency. To characterize $\sigma_{f}$ of brain tissue following electroporation-induced changes, we utilized the conductivity value at a frequency of 10 MHz ( $\sigma_{f}$ = 0.2917 S/m). This assumption, that 10 MHz mirrors the behavior of fully electroporated cells is supported by literature indicating that the β-dispersion of tissue reaches a plateau around this frequency.^19^

Additionally, $E_{\text{range}}$ and $E_{\text{delta}}$ were extrapolated from data presented by Zhao *et al.*^20^ The parameter A was determined using the following relationship:

$$A=\frac{\sigma_{f}}{\sigma_{0}}-1.$$ (7)

A comprehensive list of the parameters used for dynamic conductivity changes due to electroporation is shown in Table II.

**TABLE II.** Dynamic conductivity curve parameters. Final conductivity (*σ_f_* at 10 MHz) =0.2917.

Waveform	Conductivity *σ*_0_ (S/m)	E_range_ (V/cm)	E_delta_ (V/cm)	A (unitless)
5-5-5 µs	0.1275	350	1200	1.2878
50 kHz	0.1275	350	1200	1.2878
2-3-2 µs	0.1340	387	1232	1.1769
100 kHz	0.1340	387	1232	1.1769

Maximum voltages for both the sine (680 V for voltage matching groups and 460 V for the LD-50 kHz group) and the traditional square waves (480 V) were used to extract the electric field threshold (EFT). The EFT was backed out by determining the field contour line that best matched the volume measured from the experimental data.

### E. Statistical analysis

GraphPad Prism v10.1 (GraphPad Software, San Diego, CA) was used to conduct statistical analysis in all cases. Ordinary one-way ANOVAs with multiple comparisons were used to compare the means of all groups to one another in each set of results groups. Nonlinear fits with exponential plateaus were used to determine regression on voltage ramp data.

## SUPPLEMENTARY MATERIAL

See the supplementary material for figures regarding the validation of dynamic conductivity curves and numerical temperature monitoring, and a list of specific electrical and thermal properties for computational modeling.

## Data Availability

The data that support the findings of this study are available from the corresponding author upon reasonable request.
